# China’s progress in developing fourth-generation nuclear reactors

**DOI:** 10.1093/nsr/nwaf509

**Published:** 2025-11-22

**Authors:** Mu-ming Poo

In June 2024, a research team from the Shanghai Institute of Applied Physics (SINAP) of Chinese Academy of Sciences (CAS) announced that the 2-MW thermal thorium-based molten salt reactor (TMSR) in Wuwei, Gansu Province had achieved 100% operational capacity. Earlier last month (November 2025), the team further announced a landmark achievement: the conversion of ^232^Th into fissile ^233^U within the reactor. This breakthrough demonstrates the feasibility of using abundant thorium as a nuclear fuel for a TMSR—a cornerstone of this fourth-generation nuclear technology, which aims to deliver low-cost and sustainable energy with minimal nuclear waste.

The TMSR in Gansu is the first operational molten salt reactor globally since the USA discontinued its Oak Ridge program in 1969. It uses a thorium–uranium fuel mixture dissolved in a fluoride salt coolant, with key focuses on closed fuel cycles, online refueling and advanced waste management. Construction of the Gansu reactor began in 2018, with accelerated completion by 2022. In October 2023, it successfully reached criticality—the point at which a self-sustaining nuclear chain reaction is achieved. With the recent success in thorium-to-uranium conversion, the TMSR has moved closer than ever to a fully operational fourth-generation reactor. The scaling-up plan of the TMSR includes the development of a 100-MWe demonstration project and realizing its demonstration application by 2035. Full commercialization of TMSR technology is expected by 2040, with a dual focus on optimizing closed thorium–uranium fuel cycles and expanding non-electric applications such as hydrogen production and methanol synthesis.

The most significant strategic advantage of TMSR technology lies in the global abundance of thorium. China alone has ∼280 000 tons of thorium reserves, primarily in the Bayan Obo mine in Inner Mongolia, where thorium was previously treated as a waste byproduct. The TMSR technology transforms this underutilized resource into a strategic energy asset while significantly reducing the reliance on uranium. Molten salt reactors also possess inherent safety features: they operate at low pressure, substantially reducing explosion risks, and the fuel salt can solidify rapidly in the event of a potential accident, preventing radioactive leaks. Critically, the TMSR generates far less long-lived radioactive waste compared with traditional nuclear reactors, and the recycling of fissile materials further minimizes the waste volumes. Unlike conventional reactors, which require proximity to coastal areas or rivers for cooling, the TMSR is ideally suited for water-scarce regions such as northwestern China and central Asian countries, effectively addressing energy needs in underdeveloped areas.

The merits of the TMSR did not escape the notice of nuclear scientists worldwide, and active research and pilot projects have been ongoing in multiple countries. In the USA, TMSR development was revived by private companies such as Thorcon, which plans to build a 500-MWe TMSR in Indonesia by 2029. In 2024, Copenhagen Atomics and the UK’s National Nuclear Lab established a UK–Denmark partnership to conduct thorium criticality tests, with a specific focus on waste-burning TMSRs. Most research on TMSR in EU nations remains at the lab-scale prototype stage, concentrating on corrosion control and fuel chemistry optimization. India has also launched an ambitious thorium program via an alternate pathway, using pressurized heavy-water reactors to breed plutonium for thorium fuel cycles.

Several key factors have driven China’s progress in the TMSR program, outpacing other international efforts in advancing toward commercialization. The first is the Chinese government’s commitment to and timely investment in cutting-edge clean-energy technologies and sustained funding from CAS’ Strategic Priority Program since 2011. The second factor is the vision, audacity and perseverance of leading scientists such as the late Dr. Hongjie Xu, former SINAP director, who spearheaded the program for 16 years to overcome numerous technical barriers. The third is the remarkable cohesion and dedication of SINAP’s TMSR team—a group of hundreds of engineers and technicians who work year-round at a remote site in the Gobi Desert.

Fully functional TMSR will emerge in a timely way to help achieve China’s carbon-neutrality goal, by replacing coal-fired plants and facilitating clean hydrogen and chemical production. While actively contributing to the International Thermonuclear Experimental Reactor, China’s parallel advancement of TMSR technology underscores its ambition to develop both fusion and advanced fission technologies. China’s progress in TMSR development represents a transformative leap in fission nuclear technology, offering a safer, more sustainable energy solution that aligns with both geopolitical and environmental priorities. By leveraging domestic thorium reserves and pioneering the development of fourth-generation reactors, China is well positioned to reshape the global energy landscape and accelerate its transition to a low-carbon economy.

## Notes

Executive Editor-in-Chief, NSR

